# Stromal Caveolin-1 and Caveolin-2 Expression in Primary Tumors and Lymph Node Metastases

**DOI:** 10.1155/2018/8651790

**Published:** 2018-04-10

**Authors:** Wladimir Gerstenberger, Michaela Wrage, Eeva Kettunen, Klaus Pantel, Sisko Anttila, Stefan Steurer, Harriet Wikman

**Affiliations:** ^1^Department of Tumor Biology, University Medical Center Hamburg-Eppendorf, Hamburg, Germany; ^2^Research and Service Centre for Occupational Safety, Finnish Institute of Occupational Health, Helsinki, Finland; ^3^Department of Pathology, HUSLAB and Helsinki University Hospital, Helsinki, Finland; ^4^Institute of Pathology, University Medical Center Hamburg-Eppendorf, Hamburg, Germany

## Abstract

The expression of caveolin-1 (CAV1) in both tumor cell and cancer-associated fibroblasts (CAFs) has been found to correlate with tumor aggressiveness in different epithelial tumor entities, whereas less is known for caveolin-2 (CAV2). The aim of this study was to investigate the clinicopathological significance and prognostic value of stromal CAV1 and CAV2 expression in lung cancer. The expression of these two genes was investigated at protein level on a tissue microarray (TMA) consisting of 161 primary tumor samples. 50.7% of squamous cell lung cancer (SCC) tumors showed strong expression of CAV1 in the tumor-associated stromal cells, whereas only 15.1% of adenocarcinomas (AC) showed a strong CAV1 expression (*p* < 0.01). A strong CAV2 stromal expression was found in 46.0% of the lung tumor specimens, with no significant difference between the subtypes. Neither CAV1 nor CAV2 stromal expression was associated with any other clinicopathological factor including survival. When the stromal expression in matched primary tumors and lymph node metastases was compared, both CAV1 and CAV2 expressions were frequently found lost in the corresponding stroma of the lymph node metastasis (40.6%, *p* = 0.003 and 38.4%, *p* = 0.001, resp.). Loss of stromal CAV2 in the lymph node metastases was also significantly associated with earlier death (*p* = 0.011). In conclusion, in contrast to the expression patterns in the tumor tissue of lung cancer, stromal expression of CAV1 in primary tumors was not associated with clinical outcome whereas the stromal expression of especially CAV2 in the metastatic lymph nodes could be associated with lung cancer pathogenesis.

## 1. Introduction

Caveolin-1 (CAV1) and caveolin-2 (CAV2) are integral membrane proteins found in the outer cell membrane called caveolae and in other intracellular membranes of the endoplasmic reticulum, Golgi apparatus, and transport vesicles [1, 2]. Both proteins are also found as soluble proteins [2, 3] and are expressed in fibroblasts, endothelial cells, lipocytes, and in type I pneumocytes [4, 5]. They are involved in various mechanisms like membrane trafficking as well as signal transduction and gene regulation of cell growth and apoptosis [2, 6–8]. Caveolins have also been implicated in many processes involved in cancer initiation and metastasis [6, 9]. It has been shown that CAV1 may possess both tumor suppressing and oncogenic functions, depending on progress and tumor type [10]. In early stages of the disease, CAV1 has been shown to function mainly as a tumor suppressor, whereas at later stages, CAV1 expression has been associated with progression and metastasis [6, 11]. The role of CAV2 in carcinogenesis is less well documented. In our previous study, we analyzed the expression of both proteins in primary tumors from lung cancer patients [4]. Loss of CAV1 protein expression was detected in 48% of the tumors, whereas 28% of the tumors did not express CAV2. CAV2 protein expression correlated with shorter survival whereas no such association was found for CAV1 [4].

Nowadays, it is well-known that the tumor microenvironment plays a critical role in determining tumor initiation and progression [12]. The tumor microenvironment consists among others of stromal cells such as cancer-associated fibroblasts, which serve under normal physiological conditions as a barrier to prevent malignant transformation. However, during neoplastic transformation, the tumor-associated stroma may enable tumor progression and metastasis in response to molecular signals derived from the tumor cells [13, 14]. The level of expressed CAV1 in cancer-associated stroma has been shown to be associated with clinical parameters such as survival time. The absence of stromal CAV1 in breast cancer was shown in numerous studies to be associated with early disease recurrence, advanced tumor stage and lymph node metastases, increased distant metastasis, and poor survival [15–19]. Loss of stromal CAV1 expression has also been correlated with poor prognosis in the malignant melanoma; esophageal and oral squamous cell carcinoma; and gastric, prostate, pancreatic, and colorectal cancer [10, 20–29]. In malignant pleural mesothelioma and in tongue squamous cell carcinoma, an inverse correlation was found between CAV1 stromal expression and survival [30, 31]. The role of stromal CAV1 in the lung cancer is controversial: in two studies with small case numbers (*n* = 37 and *n* = 62), the stromal CAV1 expression was correlated with improved survival [32, 33], whereas in a large study (*n* = 412), the expression of stromal CAV1 in lung adenocarcinoma (AC) was associated with a significantly worse overall survival [34]. Only few data are found regarding the association of stromal CAV2 with tumor progression. The expression of CAV2 was shown to be raised in the stroma with the degree of invasivity of breast cancer [32]. Another study showed in thyroid cancer a correlation between stromal CAV2 expression with larger tumors, tumors with bilateral lobe involvement, and higher number of lymph node metastases (>8) [33].

To explore the relationship between stromal CAV1 and CAV2 expression and lung cancer, we analyzed expression for both proteins in stromal cells of the primary tumor as well as lymph nodes of human lung cancer tissues placed on TMAs. To our knowledge, the putative role of stromal CAV2 expression on tumor behavior in lung cancer has not been reported previously.

## 2. Methods

### 2.1. Tissue Microarrays and Patient Samples

Two different tissue microarrays (TMA) were used. The results of tumor caveolin expression from first TMA have already been reported previously [4]. Here, four replicates of tumor tissue of 1.0 mm in diameter from minimum two different paraffin blocks in each case were placed on TMA blocks in addition to two samples representing normal central bronchus and peripheral lung. Altogether, tumor samples from 161 patients were placed on these TMA. Corresponding lymph node metastases (two replicates) were also placed on the TMAs from 47 patients. The second TMA consisted of 13-matched primary tumor tissues and matched lymph node metastases as well as 4 additional unmatched lymph node samples (two replicates) [34]. Evaluable results of tumor-related stromal expression were obtained from 147 patients for CAV1 and 150 patients for CAV2. 68 of the 150 primary tumor samples were classified as squamous cell carcinoma (SCC), 51 as AC, five as AC-SCC, 10 as large-cell lung carcinoma (LCLC), eight as small-cell lung carcinoma (SCLC), four as large-cell neuroendocrine carcinoma (LCNEC), and three as giant cell carcinoma of the lung (GCCL). All patients were personally interviewed, and their consent to take part in the study and to use their tissue was obtained. The study protocol has been approved by the Ethical Review Board for Research in Occupational Health and Safety (75/E2/2001).

### 2.2. CAV1 and CAV2 Immunohistochemistry

The immunohistochemistry was published before [4]. Briefly, TMA sections (4 *μ*m) were deparaffinized and rehydrated in a series of graded alcohol. The sections were pretreated for antigen retrieval in a microwave oven in Tris-EDTA buffer (pH 9.0). The antibody staining was performed in a TechMate Horizon immunostainer (DAKO ChemMate, Denmark) using primary mouse monoclonal anti-caveolin-1 (clone 2297) and anti-caveolin-2 (clone 65) antibodies (BD Transduction Laboratories, San Jose, CA, USA). Negative controls were performed by replacing the primary antibody with buffer [4].

### 2.3. Scoring and Statistical Analyses of IHC Results

Each sample on the TMA was analyzed by two independent investigators (Wladimir Gerstenberger and Stefan Steurer). The percentage of tumor cells stained positive and the intensity of staining was recorded from each sample. An arbitrary scale of 0, 1, and 2 was used. A value of zero was given when less than 15% of the cells were positive, 1 for a weak homogenous staining (15%–60%), and 2 for an intense (high) staining (60%–100%) corresponding to the staining of stromal lung tissue was present.

Chi-square or Fisher's exact test was used to analyze the correlation between caveolin expression status and patients' clinical parameters. Patient groups were combined if the group size was below 10 cases. Survival analyses were performed using the Kaplan-Mayer method for survival curves, and survival differences were analyzed with log-rank-test using the SPSS version 20 software.

## 3. Results

### 3.1. Caveolin 1 Expression in Tumor Stroma

**Figure 1:**
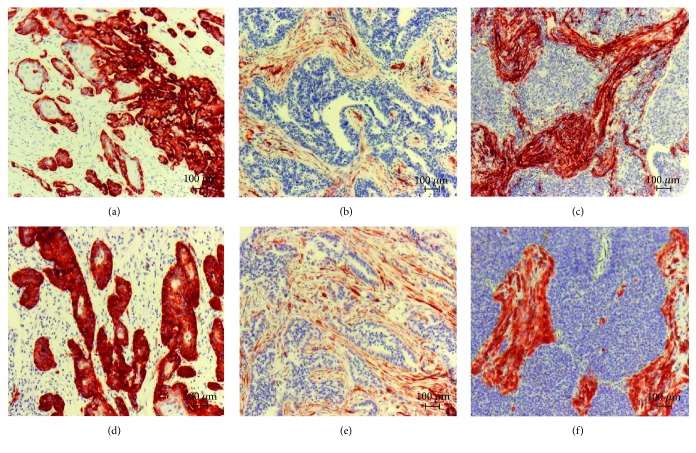
Immunohistochemical staining for CAV1 (a–c) and CAV 2 (d–f) on lung cancer tissue samples. (a + d) Negative staining of stromal cells and positive staining of tumor cells in squamous cell lung cancer patients. (b + e) Weak staining of stromal and negative staining of tumor cells in adenocarcinoma patients. (c + f) Strong positive staining of stromal cells and negative staining of tumor cells in neuroendocrine tumors.

In our study, the expressional pattern of CAV1 in the primary tumor-associated stroma was analyzable for 147 primary lung cancer cases. CAV1 expression in the stromal cells surrounding the tumors revealed positive expression in 63.9% patients (Table 1). Of the positive cases, 35.4% had a strong staining, and 28.5% had a weak staining of the stromal cells. Representative examples are shown in Figure 1.

Statistical analyses showed a significant association between level of stromal CAV1 in primary tumors with the histological type (*p* < 0.01). SCC showed frequently high levels of stromal CAV1 expression (50.7%), whereas in AC only, 15.1% of the sample showed high amounts of stromal CAV1 (Table 1). Also, patients with a large-cell lung cancer tumor showed a high stromal CAV1 expression (55.6%). Stromal CAV1 expression was not associated with any other clinicopathological factor (Table 1) even when the histological subtypes were analyzed separately (data not shown). No significant correlation between the expression levels of stromal CAV1 in primary tumors and overall survival as well as patient's outcome could be found (data not shown).

### 3.2. Caveolin 1 Expression in the Stroma of Lymph Node Metastasis

The CAV1 expression in stromal cells was also analyzed in 39 lymph node metastases samples. 29 patients (74.4%) showed no expression of CAV1 in stromal cells of the lymph nodes and 10 samples (25.6%) harbored a positive staining for CAV1. Expression of CAV1 in tumor-associated stroma and corresponding lymph node metastasis associated stroma (*n* = 32) showed concordance in 53.1% of the cases (*n* = 17) (Table 2). Two negative tumor sample showed a high expression in the stromal lymph node cells whereas 40.6% (*n* = 13) samples with an expression in the stromal tumor cells showed no expression in the lymph nodes, indicating that CAV1 is less often expressed in the corresponding stromal tissue of the lymph nodes (*p* = 0.003) (Table 2).

### 3.3. Stromal Caveolin 2 Expression in Primary Tumors and Lymph Node Metastasis

The stromal CAV2 expression was evaluable in 150 in primary lung cancer tissues. Analyses for CAV2 expression in stromal cells of the tumors revealed a positive expression in 115 patients (76.7%) and negative expression in 35 patients (23.3%) (Table 3). Of the positive cases, 46 samples (30.7%) showed a weak staining, and 69 (46.0%) showed a strong staining. Representative examples are shown in Figure 1. Similar to CAV1, the expression of stromal CAV2 around or within the primary tumor cells did not show any correlation with clinical parameters including survival. As we originally found an association of CAV2 expression in the tumor tissue [4], we also combined the results from tumor and stromal expression. No survival benefits could be detected for any group (data not shown).

Stromal CAV2 was expressed in 15 lymph node samples (34.8%) whereas 28 samples showed no expression (65.1%). Interestingly, 75.0% (*n* = 27) of the patients that died during the study period had no CAV2 expression in the stroma of lymph node metastases, whereas 83.3% of the patients who were still alive were positive for CAV2 (*p* = 0.011). Comparison between the expression of stromal CAV2 of matched tumor and lymph node metastasis samples (*n* = 39) showed a concordance in 56.4% (*n* = 22) (Table 2). Two negative tumor samples exhibit a staining of the stroma surrounding the lymph node metastasis, whereas 15 (38.4%) samples with a positive staining for CAV2 in the tumor stroma showed no staining in the stroma of lymph node metastasis (*p* = 0.001), indicating a downregulation of stromal CAV2 in the metastatic lymph nodes.

## 4. Discussion

Caveolins are known to play an important role in tumor initiation and progression. Several publications have already investigated the association between caveolin expression in primary and metastatic lung tumor tissue and cancer outcome [35–44]. In the last decades, it was shown that the cancer stroma has an important influence on tumor behavior. Whereas in the tumor tissue, high caveolin expression has usually been associated with a poor outcome; also, in other tumor entities, the inverse association has been found in cancer-associated stromal tissue [15–25].

In this study, we showed that the expression of CAV1 and CAV2 is deregulated in stromal cells of the primary tumor as well as in the stroma of lymph nodes. For both CAV1 and CAV2, a large heterogeneity in the stromal expression levels was found between different samples. Whereas some patients were totally negative for caveolins, other patients showed a very intensive stromal caveolin expression. However, no association to any clinical factor could be found for the caveolin expression in the primary tumor stroma, which is in contrast to many other tumor entities. In contrast to our current study, Bertino et al. investigated late-stage patients with poor prognosis and reported that high CAV1 stromal expression in mainly squamous cell lung cancer patients was associated with improved overall survival [42]. The study of Onion et al. demonstrated also that high stromal CAV1 is associated with improved overall survival in operable NSCLC patients [45]. Shimizu et al. on the other hand manifested the correlation between the stromal CAV1 expression in stage I lung AC and shorter recurrence-free survival [46]. We investigated mostly early-stage patients with fairly good prognosis but did not find any correlation.

Interestingly, we found in matched primary and lymph node metastases tissue a significant reduction of caveolin expression in the lymph node stroma, indicating that during the metastatic progression stromal caveolin could be lost and thus supporting the finding of a negative association between loss of caveolins and bad prognosis. The functional role of caveolin expression in the tumor stroma is still not clearly understood. Loss of caveolins in the stromal cells could be regulated by signals from metastatic tumor cells to enable the tumor cells to escape the growth-suppressing properties of the stroma and convert the normal fibroblast to cancer-associated fibroblasts (CAFs). Indeed, silencing of CAV1 in fibroblasts has been shown to induce the conversion of benign stromal fibroblasts to CAFs by the TGF-*β* pathway [22, 47, 48]. Furthermore, Bonuccelli et al. showed in a breast cancer mouse model that Cav-1-deficient stromal fibroblasts upregulate the expression of glycolytic enzymes, a hallmark of the Warburg effect, and this may provide energy-rich metabolites in a paracrine fashion to the tumor cells, supporting the clinical findings in breast cancer [49]. In lung cancer, however, the potential role of stromal caveolin expression as predictive and prognostic markers needs clearly still to be assessed in further larger studies combined with functional analyses.

## Figures and Tables

**Table 1:** Correlation between caveolin-1 expression in lung cancer stroma and clinical parameters.

	Total	Negative		Weak		Strong		*p* value
	*n*	*n*	%	*n*	%	*n*	%	
All	147	53	*36.1*	42	*28.5*	52	*35.4*	
Sex								0.25
Female	27	6	*22.2*	9	*33.3*	12	*44.5*	
Male	120	47	*39.2*	33	*27.5*	40	*33.3*	
Age								0.79
<MW	59	22	*37.3*	18	*30.5*	19	*32.2*	
>MW	83	30	*36.1*	22	*26.5*	31	*37.4*	
n.a.	5							
Histology								<0.01
SCC	69	17	*24.6*	17	*24.7*	35	*50.7*	
AC	53	29	*54.7*	16	*30.2*	8	*15.1*	
LCLC	9	3	*33.3*	1	*11.1*	5	*55.6*	
Others	16	4	*25.0*	8	*50.0*	4	*25.0*	
Grade combined								0.79
G1 + 2	66	22	*33.3*	21	*31.8*	23	*34.9*	
G3 + 4	73	28	*38.4*	20	*27.4*	25	*34.2*	
n.a.	8							
Tumor size								0.17
pT1	30	10	*33.3*	13	*43.4*	7	*23.3*	
pT2	86	31	*36.1*	21	*24.4*	34	*39.5*	
pT3	21	9	*42.9*	3	*14.2*	9	*42.9*	
pT4	8	3	*37.5*	4	*50.0*	1	*12.5*	
n.a.	2							
Lymph node status								0.09
pN0	76	32	*42.1*	22	*29.0*	22	*28.9*	
pN1	41	9	*22.0*	15	*36.6*	17	*41.4*	
pN2 + 3	28	12	*42.9*	4	*14.3*	12	*42.9*	
n.a.	2							
Distant metastasis								0.26
pM0	136	47	*35.6*	39	*28.7*	50	*36.7*	
pM1	9	5	*55.6*	3	*33.3*	1	*11.1*	
n.a.	2							
UICC stage								0.10
IA + B	70	29	*41.4*	21	*30.0*	20	*28.6*	
IIA + B	31	6	*19.4*	11	*35.4*	14	*45.2*	
IIIA+B	34	12	*35.3*	6	*17.6*	16	*47.1*	
IV	9	5	*55.6*	3	*33.3*	1	*11.1*	
n.a.	3							
Death								0.74
No	37	12	*32.4*	10	*27.0*	15	*40.6*	
Yes	107	40	*37.4*	31	*29.0*	36	*33.6*	
n.a.	3							
Smoking								0.99
Current	66	23	*34.9*	20	*30.3*	23	*34.8*	
Ex-smoker	63	24	*38.1*	20	*31.7*	19	*30.2*	
Never	3	1	*33.3*	1	*33.3*	1	*33.3*	
n.a.	15							
Asbestos exposure								0.18
Exposed	42	20	*47.6*	10	*23.8*	12	*28.6*	
Nonexposed	90	28	*31.1*	30	*33.3*	32	*35.6*	
n.a.	15							

n.a.: not available; ex-smoker: quit smoking > 6 months prior to surgery.

**Table 2:** Correlation between expressions of caveolins in tumor-associated stroma and corresponding lymph node metastasis-associated stroma.

		CAV1		CAV2	
		LN stroma^∗^		LN stroma^∗∗^	
		neg	pos	neg	pos
PT stroma	neg	12	2	10	2
	pos	13	5	15	12

^∗^ *p* value = 0.003; ^∗∗^*p* value = 0.001.

**Table 3:** Correlation between caveolin-2 expression in lung cancer stroma and clinical parameters.

	Total	Negative		Weak		Strong		*p* value
	*n*	*n*	%	*n*	%	*n*	%	
All	150	35	*23.3*	46	*30.7*	69	*46.0*	
Sex								0.41
Female	25	6	*24*	5	20	14	56	
Male	125	29	*23*	41	33	55	44	
Age								0.60
<MW	73	20	27	21	29	32	44	
>MW	74	15	20	23	31	36	49	
n.a.	3							
Histology								0.36
SCC	70	13	19	21	30	36	51	
AC	59	14	24	22	37	23	39	
LCLC	10	4	40	1	10	5	50	
Others	11	4	26	2	18	5	45	
Grade combined								0.57
G1 + 2	66	13	20	19	29	34	52	
G3 + 4	75	18	24	25	33	32	43	
n.a.	9							
Tumor size								0.86
pT1	28	7	25	8	29	13	46	
pT2	90	20	22	28	31	42	47	
pT3	22	6	27	5	23	11	50	
pT4	8	2	25	4	50	2	25	
n.a.	2							
Lymph node status								0.84
pN0	75	17	23	24	32	34	45	
pN1	45	11	24	11	24	23	51	
pN2 + 3	27	6	22	10	37	11	41	
n.a.	3							
Distant metastasis								0.76
pM0	137	32	23	42	31	63	46	
pM1	10	3	30	2	20	5	50	
n.a.	3							
UICC stage								1.00
IA + B	68	16	24	21	31	31	46	
IIA + B	36	8	22	11	31	17	47	
IIIA + B	33	8	24	10	30	15	46	
IV	10	3	30	2	20	5	50	
n.a.	3							
Death								0.61
No	36	7	19	13	36	16	45	
Yes	110	28	26	31	28	51	46	
n.a.	4							
Smoking								0.57
Current	66	19	29	15	23	32	49	
Ex-smoker	66	16	24	22	33	28	42	
None	3	0	0	1	33	2	67	
n.a.	15							
Asbestos								0.28
No	84	22	26	20	24	42	50	
Yes	47	12	26	17	36	18	38	
n.a.	19							

n.a.: not available; ex-smoker: quit smoking > 6 months prior to surgery.
